# Effectiveness and Safety of Semaglutide in Type 1 Diabetes: A Danish Nationwide Cohort Study (2018–2024)

**DOI:** 10.1016/j.lanepe.2026.101716

**Published:** 2026-05-18

**Authors:** Puriya Daniel Würtz Yazdanfard, Vanja Kosjerina, Hannah Karin Wood-Kurland, Mikkel Porsborg Andersen, Eva Hommel, Thomas Fremming Dejgaard, Kirsten Nørgaard, Christian Torp-Pedersen, Kathrine Kold Sørensen

**Affiliations:** a Copenhagen University Hospital, Steno Diabetes Center Copenhagen, Herlev, Denmark; b The Prehospital Center, Region Zealand, Næstved, Denmark; c Department of Health Science and Technology, Aalborg University, Aalborg, Denmark; d Department of Endocrinology and Nephrology, North Zealand University Hospital, Hillerød, Denmark; e Center for Clinical Metabolic Research, Gentofte Hospital, University of Copenhagen, Hellerup, Denmark; f Faculty of Health and Medical Sciences, University of Copenhagen, Copenhagen, Denmark; g Section of Biostatistics, Department of Public Health, University of Copenhagen, Copenhagen, Denmark

**Keywords:** Diabetes, Type 1, Semaglutide, Danish, Nationwide, Diabetic ketoacidosis, Hypoglycemia, HbA1c

## Abstract

**Background:**

Semaglutide is not approved for glycemic management in type 1 diabetes (T1D) due to limited evidence and safety concerns. Nevertheless, its use is increasing. We investigated glycemic and safety outcomes of semaglutide in a nationwide cohort of individuals with T1D.

**Methods:**

Using Danish registries (2018–2024), we identified individuals with T1D initiating semaglutide and matched them 1:4 using exposure density matching to unexposed control persons with T1D following them for up to two years. Glycemic trajectories were modeled using a piecewise mixed model. Cause-specific Cox models were used to estimate hospitalization rates for hypoglycemia and diabetic ketoacidosis compared with matched controls. Aalen-Johansen estimator was used to estimate adherence while accounting for death as a competing risk.

**Findings:**

We identified 879 individuals with T1D initiating semaglutide (multiple daily injections (MDI): n = 622; insulin pump: n = 257). HbA1c decreased by 5.7 mmol/mol (0.52%) (95% CI: 5.0–6.3) during the first six months and remained stable thereafter in semaglutide users while remaining unchanged in the control group. Compared with matched controls semaglutide use was not associated with an increased rate of hospitalization for hypoglycemia (HR 0.64; 95%CI: 0.35; 1.19) or diabetic ketoacidosis (HR 0.73; 95%CI; 0.34; 1.57). The cumulative probability of adherence to semaglutide at one year was 50% (95% CI: 47–54). No difference in HbA1c reduction from 0 to 6 months was observed between MDI compared to insulin pump users (P = 0.42). The median semaglutide dose redeemed during follow-up was 1.0 mg.

**Interpretation:**

In this nationwide cohort of individuals with T1D, semaglutide use was associated with no increased rate of hospitalization for hypoglycemia and no increased diabetic ketoacidosis rate, and a clinically meaningful reduction of HbA1c.

**Funding:**

This study received no funding.

**Research in context**

**Evidence before this study**

We searched PubMed from inception to Oct 1, 2025, using the following terms (“Semaglutide”[MeSH] OR semaglutide OR “GLP-1 receptor agonist∗” OR “glucagon-like peptide-1 receptor agonist∗”) AND (“Diabetes Mellitus, Type 1”[MeSH] OR “type 1 diabetes” OR T1D) NOT (“Diabetes Mellitus, Type 2”[MeSH] OR “type 2 diabetes” OR T2D). We included randomized and comparative observational studies evaluating semaglutide as adjunct to insulin in adults with type 1 diabetes. Prior evidence is very limited and consists mainly of two small, short duration trials (n = 28 and n = 78) and few observational single center chart reviews with small sample sizes. Available data suggest modest reductions in HbA1c, weight loss, and lower insulin requirements, with no clear increase in severe hypoglycemia or diabetic ketoacidosis.

**Added value of this study**

Evidence on semaglutide use in type 1 diabetes is scarce, and regulatory authorities do not recommend its use for glycemic management in this population, despite increasing real-world uptake. This nationwide registry study (n = 879) provides nationwide real-world evidence on semaglutide use in individuals with type 1 diabetes, assessing glycemic trajectories, hospitalizations for hypoglycemia and diabetic ketoacidosis, and treatment persistence in routine clinical practice.

**Implications of all the available evidence**

Taken together, available evidence suggests that semaglutide as adjunct therapy in selected individuals with type 1 diabetes may provide modest glycemic benefit and meaningful weight loss. These findings support the need for pragmatic trials to define patient selection, safety strategies, and long term outcomes.

## Introduction

Despite major advances in insulin analogs, continuous glucose monitoring, and insulin pump technology, many individuals with type 1 diabetes (T1D) still struggle to achieve recommended glycemic targets.^1^ The rising prevalence of overweight and obesity in this population further complicates glycemic management by increasing insulin resistance, daily insulin requirements, and glycemic variability.^2^, ^3^, ^4^ In addition, T1D is associated with up to a fourfold higher risk of cardiovascular disease compared with the general population, emphasizing the need for adjunctive therapies that can improve both glycemic control and cardiometabolic health.^5^

Semaglutide, a glucagon-like peptide-1 receptor agonist (GLP-1), has transformed the treatment of type 2 diabetes and obesity by improving glycemia and reducing body weight and cardiovascular risk.^6^ Among these agents, semaglutide has demonstrated greater efficacy than earlier GLP-1 analogues for both glycemic lowering and weight reduction. However, semaglutide is not approved for glycemic management in T1D.

Earlier generations of GLP-1 receptor agonists evaluated as adjunctive therapy in T1D showed modest reductions in HbA1c but raised safety concerns, including increased risks of diabetic ketoacidosis and severe hypoglycemia.^7^, ^8^, ^9^ Evidence on semaglutide in T1D remains limited to small, short-term trials and single-center chart reviews, and its real-world safety and effectiveness on a population level are completely unknown.^10^, ^11^, ^12^ Despite this lack of evidence, semaglutide is increasingly used among individuals with T1D.^13^

To address this evidence gap, we conducted a nationwide register study in Denmark to examine glycemic trajectories and safety outcomes, including risks of hospitalization for hypoglycemia and diabetic ketoacidosis, among individuals with T1D initiating semaglutide.

## Methods

### Setting and data sources

The population for this study was identified using the Danish nationwide registries, which provide comprehensive data on all residents in Denmark. All Danish residents are assigned a unique Civil Personal Registration (CPR) number at birth or upon immigration, enabling linkage across multiple health and administrative databases at an individual level.^14^ Healthcare in Denmark is tax-funded and free for all citizens, covering both general practitioners and hospital care. Medication costs are partly covered by patients through out-of-pocket expenses, although the majority of these costs are reimbursed for most medications. However, off-label prescriptions, such as the use of semaglutide in individuals with T1D, are not covered by these reimbursement schemes.

Data for this study were retrieved from five nationwide registries. The Danish National Patient Registry contains information on all inpatient and outpatient hospital contacts since 1977, coded using the International Classification of Diseases (ICD).^15^ The Danish National Prescription Registry tracks all prescriptions redeemed at pharmacies since 1995, including dispensing dates and Anatomical Therapeutic Chemical (ATC) codes.^16^ Mortality data were sourced from the Danish Civil Registration System.^17^ Biomarker data, including HbA1c measurements, were retrieved from the Clinical Laboratory Information System Research Database.^18^ Data on body mass index (BMI) in patients with diabetes, were retrieved from the Danish Adult Diabetes Registry.^19^ For this study, all registries used contained updated information through January 2024, except for the Danish Adult Diabetes Registry, which was only updated until June 2022.

### Ethics

In Denmark, register-based studies that are conducted for the purpose of statistics and scientific research do not require ethical approval or informed consent. This study was in accordance with the Danish Data Protection Act and the General Data Protection Regulation approved by the data-responsible organisation, the Capital Region of Denmark. Due to data protection regulations at Statistics Denmark, reporting of cell counts fewer than three individuals is not permitted. In such instances, categories were aggregated to comply with confidentiality requirements.

### Study period and duration

The study period spanned from January 1, 2018 to January 1, 2024. The inclusion period ended on June 1, 2023 to ensure at least six months of potential follow-up before administrative censoring on January 1, 2024. Participants were followed from time zero until the outcome of interest, death, end of data availability, or a maximum of two years of follow-up. Follow up was restricted to two years, as prespecified prior to analysis to ensure meaningful follow up and to avoid data driven extension of analyses. Historical registry information dating back to 1995 was used to define the study population.

### Cohort definition and time zero

**Fig. 1:**
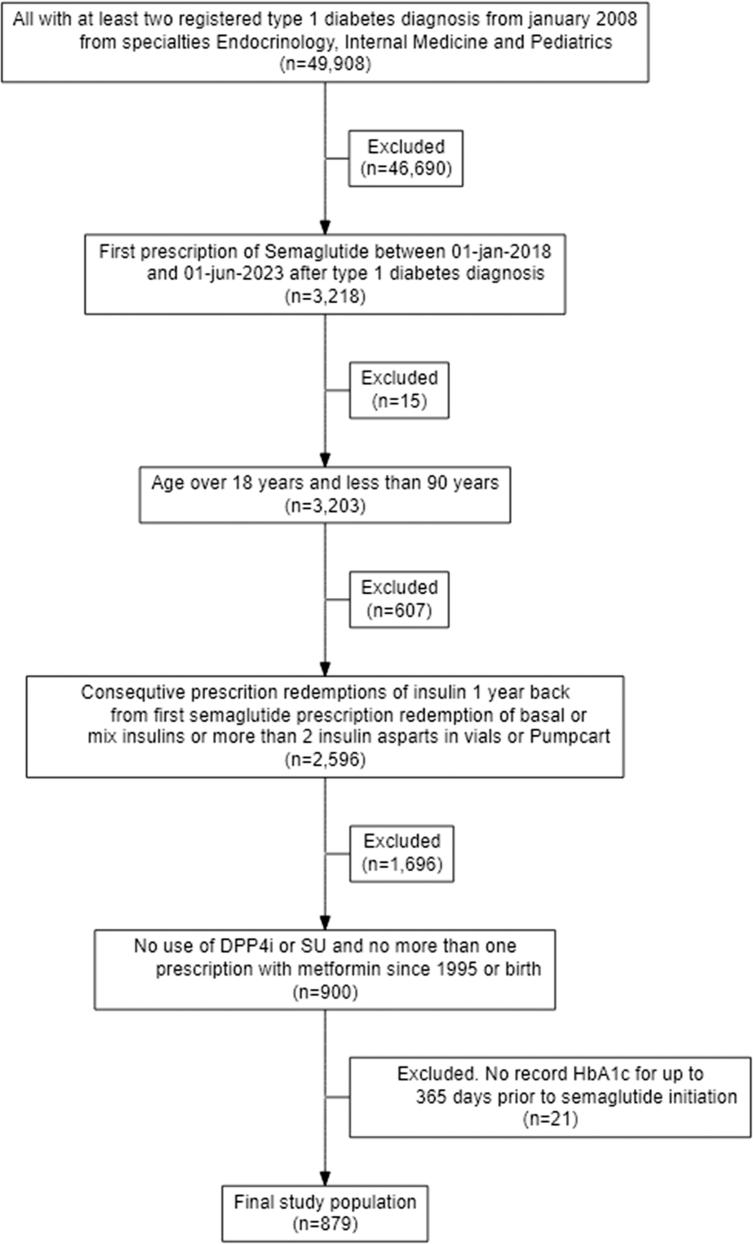
Flowchart of cohort selection.

Because T1D cannot be reliably identified by diagnosis codes alone, we applied a restrictive algorithm for defining the study population.^20^ First, we identified all individuals with at least two T1D diagnosis codes (ICD-10: DE10) registered in contacts with departments in endocrinology, internal medicine, or pediatrics. Second, we included only those who at some point redeemed a prescription for semaglutide. Third, we required that, in the three years preceding semaglutide initiation, individuals had redeemed insulin prescriptions at least twice per year, with no gap longer than 182 days. Eligible prescriptions were of either 1: long-acting insulin, 2: mixed insulin, or 3: insulin aspart in vials or PumpCart®, the latter being classified as pump use. Finally, we excluded individuals who had ever redeemed prescriptions for DPP-4 inhibitors or sulfonylureas or more than one prescription for metformin since the beginning of the prescription registry from 1995 or birth. The date of first semaglutide redemption was defined as time zero (Fig. 1).

### Exposure and adherence

Individuals were considered adherent while prescriptions covered ongoing semaglutide use, and were classified as non-adherent once 60 days had passed after the calculated duration of the prescription. The duration of each prescription was calculated as the number of packages multiplied by the number of injectable devices per package, multiplied by four doses per device, and then multiplied by seven days, corresponding to the number of days covered by each dose.

### Outcomes

The primary outcome was change in HbA1c after semaglutide initiation, modeled in two time segments. The first segment captured change from baseline to 6 months, and the second captured change from 6 to 24 months. The primary outcome was estimated in individuals with at least one HbA1c measurement during follow-up, remaining adherent to semaglutide at each HbA1c measurement date.

The secondary safety outcomes were time to first hospitalization for hypoglycemia and diabetic ketoacidosis (based on ICD-10 codes Supplementary Table S1) after semaglutide initiation, regardless of adherence.

### Variables of interest

Baseline HbA1c was defined as the most recent measurement obtained within 12 months before initiation of semaglutide. All subsequent HbA1c values recorded during periods of semaglutide use were included as follow-up measurements. Baseline BMI measurements were included if they were recorded at any given time prior to time zero (Supplementary Table S2).

Hospitalization for hypoglycemia or ketoacidosis prior to time zero was defined as having at least one hospital contact of any duration due to hypoglycemia, or a hospitalization lasting more than 12 h with a ketoacidosis diagnosis (Supplementary Table S1), respectively. Both variables were categorized as never or present if an event had occurred within 10 years prior to initiation of semaglutide. Diagnoses for diabetic ketoacidosis were disregarded if they occurred within 3 months of the first ever recorded diagnosis establishing T1D.

### Comparator group

For the analysis of safety and glycemic outcomes, we constructed a matched comparator cohort of individuals with T1D (from specialties endocrinology, internal medicine and pediatrics as above) who had not received semaglutide. Using a 1:25 ratio, exposed cases were matched to unexposed controls by birth year and sex through exposure density sampling and giving controls a matched index date of the exposure date of the case. Hereafter, the controls were filtered through the same algorithm as the cases but with their matched index date as time zero. Finally, controls were selected using k-nearest neighbors matched on BMI, HbA1c, previous hospitalization for hypoglycemia and ketoacidosis, ensuring a final consistent 1:4 ratio of cases and controls. In cases with missing BMI, we assumed the mean value of BMI among cases.

### Sensitivity and additional analysis

To evaluate glycemic outcomes irrespective of treatment adherence, we conducted a sensitivity analysis in which all individuals were followed from semaglutide initiation regardless of subsequent discontinuation and we did analysis with 30 and 90 day adherence grace periods. Total insulin prescription redemption was calculated as the cumulative redemption of milliliters of insulin, irrespective of type, 180 days prior to time zero and day 90 to day 270 after time zero (180 days total cumulative dose).

In addition, analyses were performed for secondary safety outcomes restricted to cases with a BMI measurement. Finally, addressing missing BMI, a sensitivity analysis using multiple imputation by chained equations with predictive mean matching was performed.^21^ The imputation model included baseline variables; age, sex, HbA1c, prior hypoglycemia, prior ketoacidosis, myocardial infarction, stroke, heart failure, renal disease, and diabetic nephropathy. A total of 50 imputed datasets were generated. Matching was repeated within each imputed dataset and estimates were pooled using Rubin’s rules.^22^

### Statistical analysis

HbA1c trajectories were analyzed using linear mixed-effects models with random intercepts for each participant to account for repeated measurements among those with a HbA1c measurement during follow-up. Time was modeled in two piecewise linear segments, representing change from baseline to six months and change beyond six months. Fixed effects included age, sex and insulin pump use. Random intercepts allowed subject-specific baseline HbA1c values, while slopes were assumed to be common across participants. Interaction terms between the 0–6 month time segment and insulin pump use, and between the 0–6 month time segment and baseline BMI, were included. HbA1c values were predicted based on the model. Furthermore, as a sensitivity analysis, we fitted a shared random effects joint model linking longitudinal HbA1c trajectories with time to death to assess potential informative dropout.

For the secondary outcomes of hospitalization for hypoglycemia and diabetic ketoacidosis, cause-specific Cox proportional hazards models were fitted for each cause and all-cause mortality, adjusted for baseline HbA1c, age, insulin pump use and prior hospitalization for hypoglycemia or diabetic ketoacidosis. Adjusted cumulative incidence curves were subsequently derived using parametric G-computation, incorporating all-cause mortality as a competing risk. Time to semaglutide treatment discontinuation was analyzed using the Aalen-Johansen estimator with all-cause mortality as a competing risk. Where appropriate a paired t-test was used to assess differences in means for insulin consumption. All analyses were conducted in R version 4.4.3. Two-sided P-values < 0.05 were considered statistically significant.

### Role of funding source

This study received no funding

## Results

We identified 879 individuals with T1D who initiated semaglutide. Of these, 257 (29%) used insulin pumps and 622 (71%) were treated with multiple daily injections (MDI). At time zero, insulin pump users were on average 7 years younger than MDI users (P < 0.001), predominantly women (Insulin pump: 74% MDI: 49%; P < 0.001) and had 3.8 mmol/mol lower HbA1c levels (P < 0.001). BMI was missing in approximately 59% of the study population due to incomplete registry coverage. Matched cases and controls are shown in Table 1 and full baseline characteristics in Table 2.

**Table 1:** Table of controls and cases used in analysis.

Characteristic	Semaglutide, N = 879^a^	Controls, N = 3,516^a^
Sex		
Female	492 (56%)	1968 (56%)
Male	387 (44%)	1548 (44%)
Age (years)	48.0 (25.0–71.0)	48.0 (24.0–70.0)
Baseline HbA1c (mmol/mol)	63.0 (13.0)	61.3 (13.0)
Treatment type		
Multiple daily injections	622 (71%)	2654 (75%)
Insulin pump	257 (29%)	862 (25%)
BMI (kg/m^2^)	32.1 (6.0)	30.5 (2.7)
Missing	520	0
Prior hospitalization for hypoglycemia		
Last 10 years	113 (13%)	421 (12%)
No	766 (87%)	3095 (88%)
Prior hospitalization for diabetic ketoacidosis		
Last 10 years	72 (8.2%)	272 (7.7%)
No	807 (92%)	3244 (92%)
History of myocardial infarction	19 (2.2%)	45 (1.3%)
History of stroke	19 (2.2%)	30 (0.9%)
Heart failure	11 (1.3%)	24 (0.7%)
Renal disease	32 (3.6%)	91 (2.6%)
Diabetic nephropathy	14 (1.6%)	43 (1.2%)

All are selected based on the algorithm for type 1 diabetes. Hospitalisation for hypoglycemia and diabetic ketoacidosis are events prior to baseline. BMI = Body mass index. Continuous values are presented as mean (standard deviation). Age is presented with median (5–95%).

a n (%); Median (5%–95%); Mean (SD).

**Table 2:** Baseline characteristics of the Danish type 1 diabetes cohort starting semaglutide from 2018 to June 2023.

Characteristic	MDI, N = 622^a^	Insulin pump, N = 257^a^
Sex		
Female	302 (49%)	190 (74%)
Male	320 (51%)	67 (26%)
Age (years)	50.0 (27.0–72.0)	43.0 (22.8–61.0)
Baseline HbA1c (mmol/mol)	64.1 (13.2)	60.3 (12.2)
BMI (kg/m^2^)	32.2 (6.1)	31.5 (5.6)
missing	344	176

MDI = multiple daily injections. Body Mass Index (BMI). Continuous values are presented as mean (standard deviation). Age is presented with median (5–95%).

a n (%); Median (5%–95%); Mean (SD).

### Adherence and dosage

**Fig. 2:**
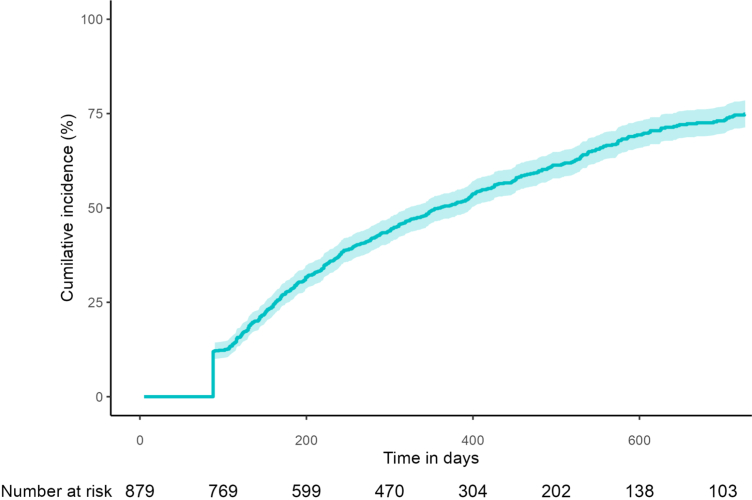
Time to non-adherence from first prescription of semaglutide among patients with Type 1 diabetes. Non-adherence was defined as the first day occurring >60 days after the expected end of supply of the last redeemed semaglutide injection, as inferred from prescription pen redemptions.

At 6 months, 71% (95% CI: 69–74) of individuals remained adherent to semaglutide. Adherence declined to 50% (95% CI: 47–54) at 12 months, and to 25% (95% CI: 22–29) by the end of follow-up at 24 months (Fig. 2). The maximum redeemed prescription dose in the study period among individuals picking up more than one prescription (n = 754) was as follows (mean dose = 0.92 mg; median dose = 1 mg): 0.25 mg (n = 32); 0.5 mg (n = 170); 1.0 mg (n = 572); 1.7 mg (n = 28); 2.4 mg (n = 17).

### Primary outcome: HbA1c trajectories

**Fig. 3:**
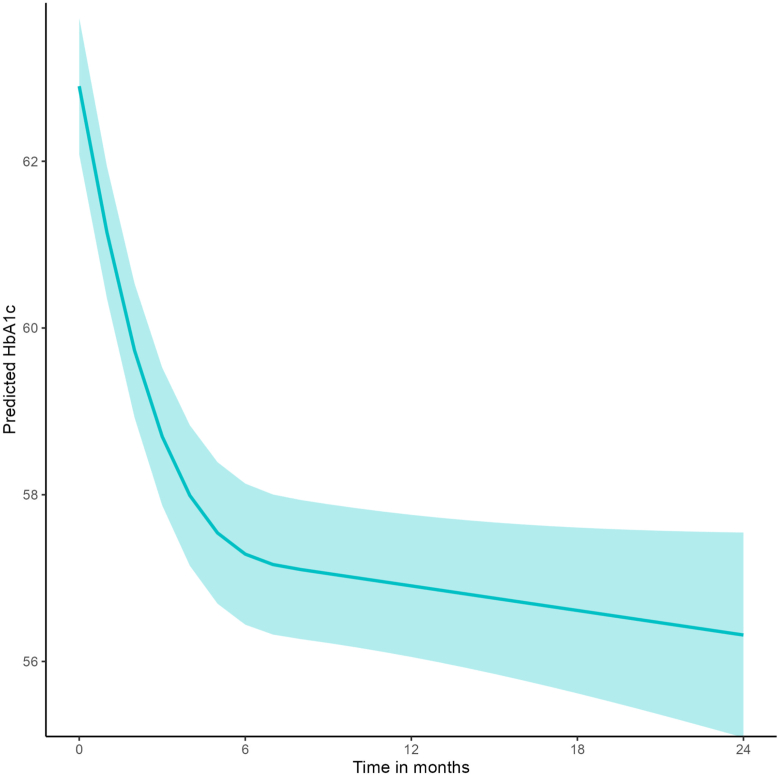
HbA1c after initiation of semaglutide in a population with Type 1 diabetes, during the period they remain adherent to semaglutide. Predicted values from a mixed model with a spline.

Mean baseline HbA1c was 63 mmol/mol (7.9%). A total of 708 (80.5%) individuals had HbA1c measurements post baseline, contributing to trajectories. During the first 6 months after initiation, HbA1c declined by 5.7 mmol/mol (0.52%) in total (95% CI: 5.0–6.3). From 6 to 24 months HbA1c remained stable, with no further significant change (slope −0.03 mmol/mol per month, 95% CI: −0.09 to 0.04) (Fig. 3). The estimated HbA1c slope from 0 to 6 months did not differ significantly between insulin pump users (6.2 [0.57%] mmol/mol; 95% CI: 5.3–7.0) and MDI users (5.6 [0.51%] mmol/mol; 95% CI: 5.0–6.3) (P = 0.42). Among controls HbA1c remained stationary from 0 to 6 months at −0.09 mmol/mol (95% CI: −0.49 to 0.31 mmol/mol).

### Secondary outcome: hypoglycemia, diabetic ketoacidosis, and mortality

**Fig. 4:**
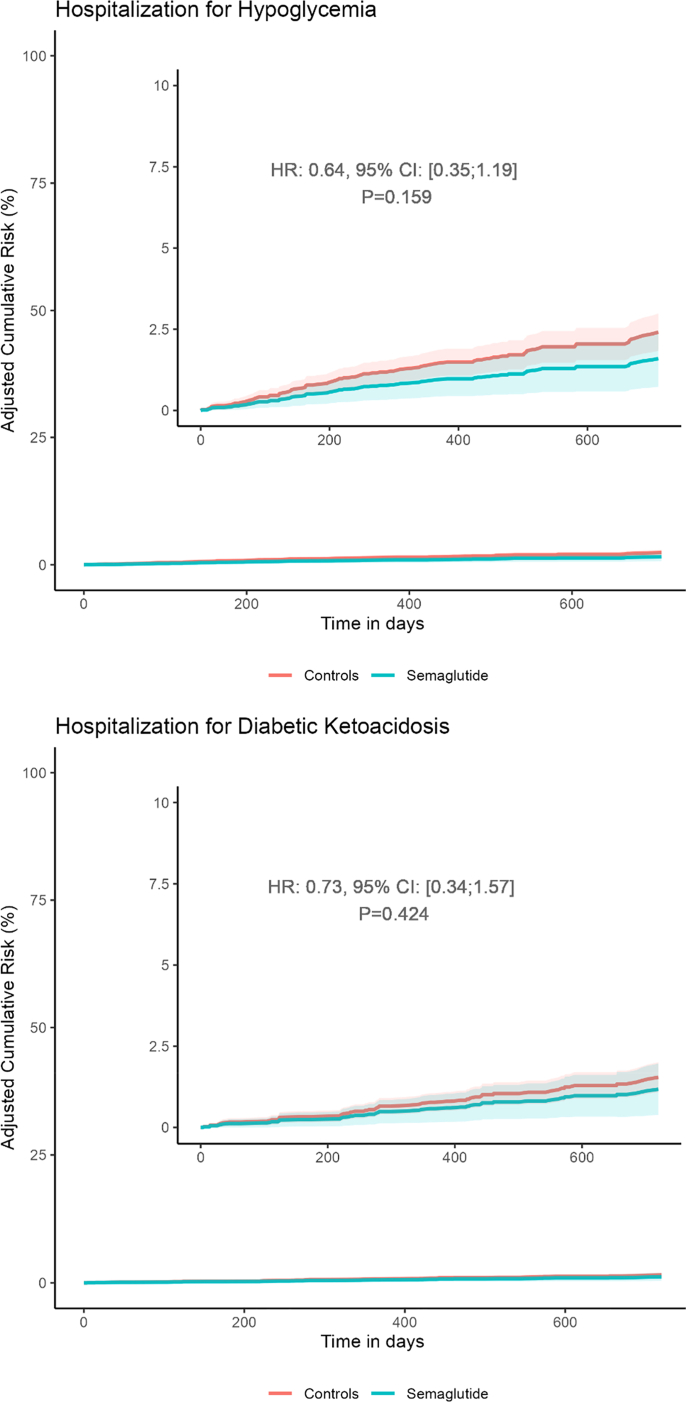
Adjusted cumulative risks of hospitalization for hypoglycemia and diabetic ketoacidosis. Adjusted for age, sex, baseline HbA1c, previous hospitalisation for diabetic ketoacidosis and hypoglycemia among patients with type 1 diabetes starting treatment with semaglutide and controls matched on age, sex, body mass index and prior hospitalization for hypoglycemia and ketoacidosis.

Semaglutide use was associated with no increased risk of hospitalization for hypoglycemia compared to controls (Hazard ratio: 0.64 (95% CI: 0.35–1.19) P = 0.16). For hospitalization due to diabetic ketoacidosis, we estimated no increased risk among semaglutide users compared to controls (Hazard ratio: 0.73 (95% CI: 0.34–1.57) P = 0.42) (Fig. 4). We observed no difference in all-cause mortality between the groups (Hazard ratio: 0.49, (95% CI: 0.22–1.09) P = 0.08). A total of 9 deaths occurred in the exposed group and 49 in the control group (1:4 ratio). Causes of death were attributed to cardiovascular disease (5 cases, 21 controls), diabetes-related complications (10 controls), and other or unknown causes (18 controls). Among cases, diabetes related and other causes were aggregated (n = 4) in accordance with data protection regulations.

### Sensitivity analysis

In individuals starting semaglutide irrespective of adherence, findings were consistent but attenuated. HbA1c declined by 4.4 mmol/mol (0.40%) during the first 6 months (95% CI: 3.8–4.9) with a slight increase thereafter (slope increase of 0.06 mmol/mol per month, 95% CI: 0.01–0.10). Analyzing safety outcomes in only complete cases with BMI did not materially change results (Hospitalization for hypoglycemia HR: 0.83 95% CI: 0.37–1.90 and hospitalization for diabetic ketoacidosis HR: 0.64, 95% CI: 0.19–2.13). Similarly after performing multiple imputations of missing baseline BMI, the hazard ratio was 0.70 (95% CI: 0.37–1.32) for hypoglycemia and 0.70 (95% CI: 0.32–1.52) for ketoacidosis. The association parameter linking HbA1c to the hazard of death was small (α = 0.047; 95% CI: −0.019 to 0.101), suggesting limited evidence of informative dropout due to death. Estimates of HbA1c trajectories were materially unchanged compared with the primary linear mixed-effects analyses. Alternate grace periods for semaglutide adherence resulted in similar HbA1c decline from 0 to 6 months (30 days: 5.9 mmol/mol; 95% CI: 5.1–6.7 and 90 days 5.6 mmol/mol; 95% CI: 4.9–6.1).

### Additional analysis

Total insulin prescription redemption declined among initiators of semaglutide from 90.5 ml to 83.1 ml (Difference of 7.4 ml 95% CI 3.6–11.2). For controls insulin prescription redemptions increased from 83.2 ml to 86.7 ml (difference of 3.5 ml. 95% CI: 1.2–5.8).

HbA1c declined from 0 to 6 months for all individuals despite baseline HbA1c categorisation, but the decline was greater among individuals with higher baseline HbA1c. For individuals with <53 mmol/mol (7.0%) at baseline the decline was 1.9 mmol/mol (95% CI: 1.1–2.8) while for ≥ 53 mmol/mol the decline was 6.8 mmol/mol (95% CI: 6.1–7.6). Likewise for <63 mmol/mol (7.9%) the decline was 3.6 (95% CI: 3.0–4.2) and for ≥ 63 the decline was 8.5 mmol/mol (95% CI: 7.5–9.6). We found no interaction between baseline BMI (among complete cases) and change in HbA1c from 0 to 6 months (P = 0.67).

Stratification by maximum achieved semaglutide dose showed greater HbA1c reductions with increasing dose, except at the lowest dose level. The estimated HbA1c changes were 8.9 mmol/mol (95% CI: 2.8–14.8) for 0.25 mg (n = 32), 4.9 mmol/mol (95% CI: 3.3–6.4) for 0.5 mg (n = 170), 6.4 mmol/mol (95% CI: 5.6–7.1) for 1.0 mg (n = 572), 7.7 mmol/mol (95% CI: 2.9–12.3) for 1.7 mg (n = 28), and 8.7 mmol/mol (95% CI: 4.8–12.7) for 2.4 mg (n = 17).

## Discussion

In this nationwide cohort, initiation of semaglutide was associated with a clinically relevant reduction in HbA1c during the first six months, which was maintained over two years of follow-up. Importantly, semaglutide use was not associated with an increased risk of hospitalization for diabetic ketoacidosis and hospitalization for hypoglycemia compared with matched controls.

This real-world evidence extends on existing smaller trial data. A randomized crossover study of 28 adults over 11 weeks with T1D reported improved glycemic outcomes and weight reduction with semaglutide.^10^ Likewise a 26-week double-blind trial in 72 adults with T1D and obesity similarly found lower HbA1c, greater time in range, and weight loss compared with placebo with no significantly increased risk of ketoacidosis and hypoglycemia.^11^ Beyond trials, few chart reviews exist aligning with our findings showing glycemic benefits when semaglutide is used in T1D.^12^

Concerns by the Food and Drug Administration (FDA) and the European Medicines Agency (EMA) have been raised regarding the risk of diabetic ketoacidosis and hypoglycemia when semaglutide is used in individuals with T1D.^23^^,^^24^ Accordingly, all formulations of semaglutide remain not indicated for the treatment of glycemia in T1D. In our nationwide study however, we found no evidence of increased risk of diabetic ketoacidosis and risk of hospitalization for severe hypoglycemia compared with matched controls. It is possible that individuals prescribed semaglutide represent a subgroup with inherently lower risk of these adverse events, despite matching and adjusting models. Additionally, these findings are confounded by the lack of data on BMI on a subset of semaglutide users.

We observed a modest reduction in redeemed insulin volume following semaglutide initiation, suggesting a potential insulin sparing effect. HbA1c reductions were more pronounced among individuals with higher baseline HbA1c. Numerically greater glycemic reductions were observed at higher achieved semaglutide doses, although small numbers limit inference regarding dose response. Stratification by maximum achieved dose reflects cumulative treatment exposure rather than the incremental glycemic effect of dose increase. As individuals initiate treatment at lower doses and titrate over time, the majority of HbA1c reduction may occur early during treatment, with more modest additional reductions following dose increases.^25^

Nevertheless, our findings suggest that in selected individuals semaglutide use may confer substantial improvements in glycemic control without increasing the risk of severe hypoglycemia. This may reflect improved glycemic stability and fewer insulin dose fluctuations following semaglutide initiation.^26^^,^^27^

Adherence to semaglutide declined substantially over time, with just over half of users remaining on treatment after one year. This is consistent with previous reports in type 2 diabetes and obesity, where gastrointestinal side effects and tolerability issues often limit long-term use.^28^^,^^29^ Additionally, the decline in adherence may partly reflect individuals using semaglutide primarily for weight loss and discontinuing treatment once their target weight was achieved. Finally, because semaglutide is not reimbursed for T1D in Denmark, the required out-of-pocket payment likely further limits long-term adherence.

The potential role of GLP-1 RA in T1D extends beyond glucose control. In Type 2 diabetes, these agents have consistently demonstrated cardiovascular and renal benefits that appear partly independent of their glucose-lowering effects.^6^ In both diabetes and non-diabetes populations GLP-1 RAs have consistently demonstrated substantial reductions in body weight. Our findings that semaglutide use in T1D was associated with improved glycemic control, no increased risk of hospitalizations for diabetic ketoacidosis and hypoglycemia are reassuring and suggest that semaglutide may be safely used in this population. If similar cardiovascular and renal benefits are confirmed in T1D, semaglutide could represent a promising adjunct therapy targeting both metabolic control and long-term complication risks.

Strengths of our study include the use of nationwide data with virtually complete coverage of prescriptions, hospitalizations, and laboratory results, minimizing selection bias and loss to follow-up. The restrictive definition of T1D, requiring sustained insulin use, increased diagnostic specificity, however potentially also omits individuals who wrongly initiate and continue on metformin for a period due to initial misdiagnosis. Our risk-set matched design and adjustment for baseline HbA1c, pump use, age and prior events reduced confounding by indication, although residual confounding from unmeasured factors, such as BMI in those missing, specific types of insulin delivery pumps with or without continuous glucose monitoring, cannot be excluded.

Due to the lack of sufficient BMI data after initiation of semaglutide, we could not estimate BMI changes, or assess semaglutide effectiveness across BMI strata. Additionally, missing BMI data in cases resulted in assuming the mean BMI for this population in order to match controls, which could result in a bias. Moreover, despite matching and covariate adjustment, individuals prescribed semaglutide may represent a subgroup with higher engagement in diabetes management, potentially introducing a “healthy user” bias, which could have affected lower rates of hypoglycemia and hospitalization due to diabetic ketoacidosis.

Moreover, semaglutide is not reimbursed for type 1 diabetes in Denmark and may be used by individuals with greater resources. In addition, lack of information on diabetes duration and some individuals excluded with missing HbA1c data may have introduced residual bias. Factors such as pump use and insulin dosing could change during follow up but were not modeled time varyingly, which may introduce residual confounding. We did not have information on automated insulin delivery systems or CGM use, which could result in bias. Despite matching and adjustment, residual and unmeasured confounding cannot be excluded. Adherence was based on prescription redemptions and may be subject to misclassification, although results were robust to alternative grace period definitions. Finally, our reliance on hospital registry data precluded assessment of non-severe hypoglycemia, everyday ketoacidosis events, CGM-derived glycemic metrics, and identification of euglycemic ketoacidosis.

### Conclusion

In this nationwide study, we found that in individuals with T1D, semaglutide was associated with no increased rate of hospitalization for hypoglycemia and no increased rate of hospitalization for ketoacidosis. Additionally, semaglutide resulted in meaningful improvements in HbA1c levels. While regulatory agencies caution against its use in T1D due to potential safety concerns, our findings suggest that, in selected individuals, semaglutide may offer meaningful clinical benefits when used as an adjunct to insulin therapy.

## Contributors

PDWY drafted the manuscript. All authors contributed to review and conceptualization of the study. PDWY, CTP, KKS, MPA, and VK had data access and verified methodology.

## Data sharing statement

Data is accessed through a safe environment on protected servers on Statistics Denmark. Access to data is available through affiliations with research departments that collaborate with Statistics Denmark.

## Declaration of interests

**TFD** serves as an advisor to Boehringer Ingelheim, Eli Lilly, Medtronic and Novo Nordisk; has received research support from AstraZeneca and Novo Nordisk; and has received speaking fees from AstraZeneca, Bayer, Boehringer Ingelheim, Novo Nordisk and Eli Lilly.

**KN** serves on behalf of her institution, as an adviser to MiniMed, Tandem, Abbott Diabetes Care, Convatec, and Novo Nordisk; owns stocks in Novo Nordisk; has received research grants to the institution from Novo Nordisk, Zealand Pharma, Dexcom, and MiniMed; and has received fees for speaking from MiniMed, Abbott Diabetes Care and Novo Nordisk.

**CTP** reports grants for studies from Novo Nordisk and Bayer.
