# Supplementary material for: Effectiveness and Safety of Semaglutide in Type 1 Diabetes: A Danish Nationwide Cohort Study (2018–2024)
Source: Lancet Reg Health Eur. 2026 May 18;66:101716. doi: 10.1016/j.lanepe.2026.101716 (PMC13208826; doi:10.1016/j.lanepe.2026.101716)
Supplement: Supplementary Figs. S1 and S2 and Tables S1 and S2 [file mmc1.docx]

## Supplementum

| **Category** | **Subcategory / Description** | **Codes / Item Numbers** |
| --- | --- | --- |
| **Diagnosis codes** | Diabetes Mellitus type 1 | DE10 |
|  | Hypoglycemia | DE159, DE159B, DE160, DE161B, DE161, DE162, DT383A |
|  | Ketoacidosis | DE101, DE111, DE121, DE131, DE141 |
| **Medication item numbers** | Insulin vials | 048328, 140619, 157540, 448253, 478728, 199965, 002208 |
|  | Insulin pump cartridge | 482486, 542479 |
|  | Semaglutide (Wegovy) | 164303, 178307, 418253, 431609, 448583, 395234, 396934, 481461, 534716, 538908, 560545, 058815, 058102, 128420, 131824, 191243, 394103, 405773, 066923, 187574, 191797, 409687, 437102, 153271, 178249, 386270, 409364, 468849, 541921 |
|  | Semaglutide (Ozempic) | 032822, 110341, 144807, 178275, 435625, 500302, 150408, 183356, 408233, 417633, 417977, 111367, 046216, 072557, 502065, 505084, 586531, 592242, 063436, 081586, 089980, 126158, 185998, 582611, 397603, 452640, 099045, 183727, 195994, 504236, 545670 |
| **ATC codes (diabetes medications)** | Insulin (all) | A10A |
|  | Insulin – bolus | A10AB |
|  | Insulin – basal | A10AC, A10AE04, A10AE05, A10AE06 |
|  | Insulin – mix | A10AD |
|  | Metformin | A10BA02, A10BD02, A10BD03, A10BD05, A10BD07, A10BD08, A10BD10, A10BD11, A10BD13, A10BD14, A10BD15, A10BD16, A10BD17, A10BD18, A10BD20, A10BD22, A10BD23, A10BD25, A10BD26, A10BD27 |
|  | Sulfonylurea | A10BB, A10BD01, A10BD02, A10BD04, A10BD06 |
|  | DPP-4 inhibitors | A10BH, A10BD07, A10BD08, A10BD09, A10BD10, A10BD11, A10BD12, A10BD13, A10BD18, A10BD19, A10BD21, A10BD22, A10BD24, A10BD25, A10BD27 |
|  | Semaglutide | A10BJ06 |

Supplementary table 1

| **Semaglutide (n = 879)** | | | | |
| --- | --- | --- | --- | --- |
| prior to baseline | 1st Quartile | Median | 3rd Quartile | Missing |
| 2 years | 28.7 | 32.5 | 36.7 | 637 |
| 5 years | 28.7 | 32 | 36.5 | 590 |
| 10 years | 28.4 | 31.7 | 36.1 | 552 |
| maximum | 27.7 | 31.1 | 35.1 | 520 |

| **Controls (n = 3516)** | | | | |
| --- | --- | --- | --- | --- |
| prior to baseline | 1st Quartile | Median | 3rd Quartile | Missing |
| 2 years | 29.1 | 30.8 | 32.1 | 1415 |
| 5 years | 29.1 | 30.8 | 32.1 | 1066 |
| 10 years | 29.0 | 30.7 | 32.1 | 570 |
| maximum | 29.0 | 30.5 | 32.0 | 0 |

Supplementary table 2: Baseline body mass index (BMI) depending on how many years back from baseline BMI data is obtained from. Coverage from 2005-2023.

**Cox model evaluation:
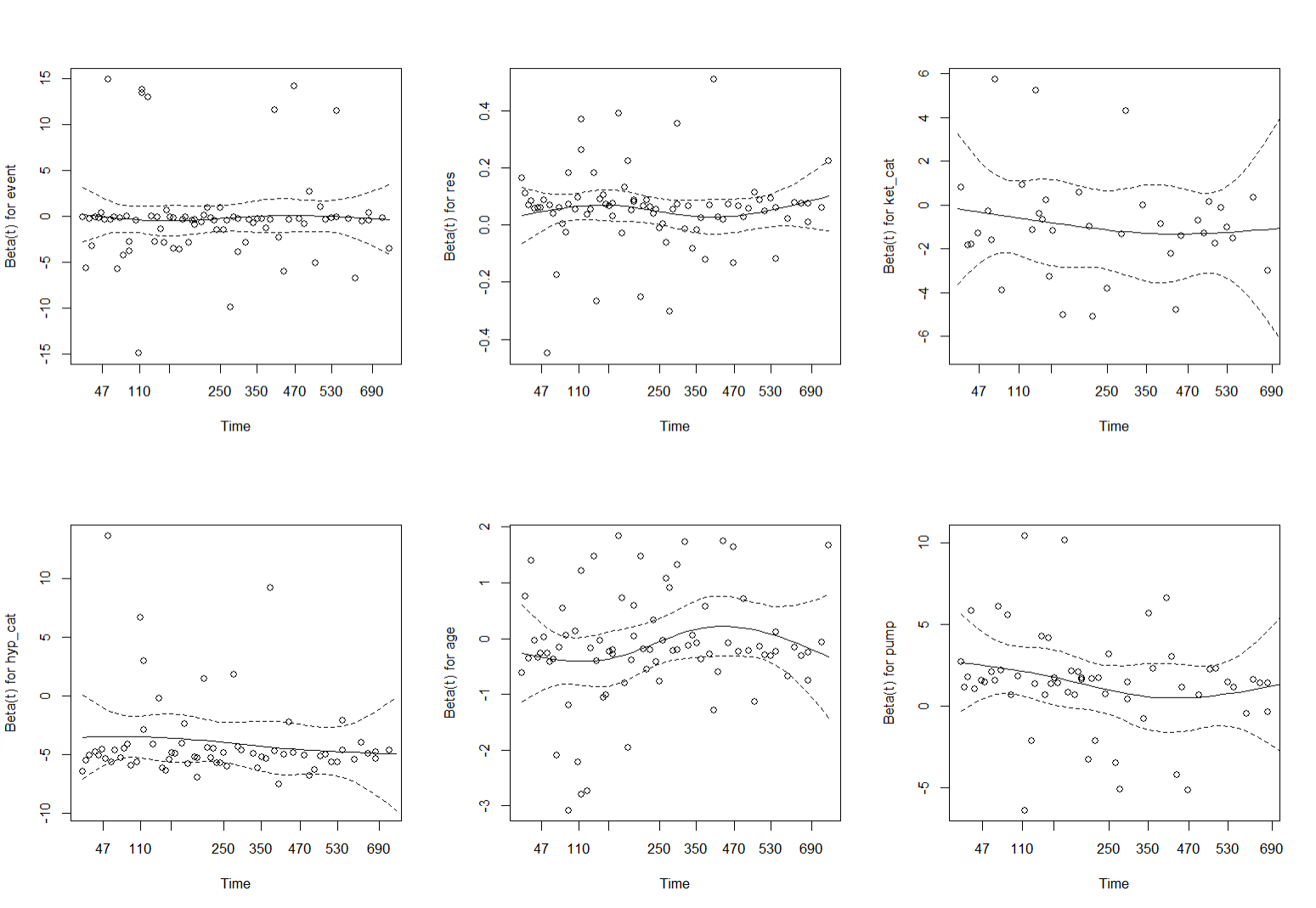
**

Supplementary figure 1: Hospitalisation for hypoglycaemia

Schoenfield Residuals scaled. Global P-value 0.41

Supplementary figure 2: Hospitalisation for diabetic ketoacidosis
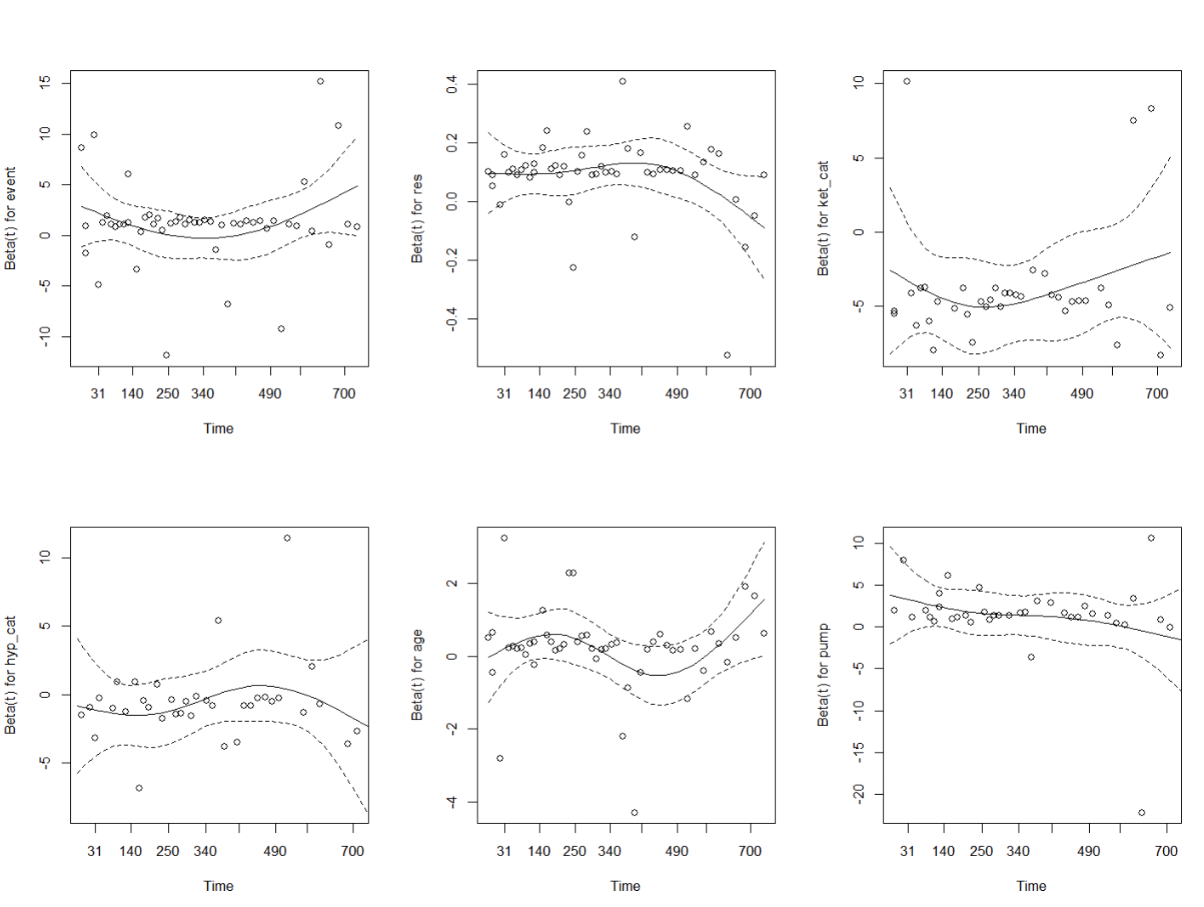


Schoenfield Residuals scaled. Global P-value 0.57
